# *APOE4* driven splicing defects disrupt neurite projection in excitatory neurons

**DOI:** 10.1002/alz70855_099084

**Published:** 2025-12-23

**Authors:** Alexandre Pelletier, Lu Qian, Tony Tuck, Julia TCW

**Affiliations:** ^1^ Boston University School of Medicine, Boston, MA, USA; ^2^ Boston University Chobanian & Avedisian School of Medicine, Boston, MA, USA

## Abstract

**Background:**

The allele e4 of the apolipoprotein E gene (*APOE4*), present in around 20% of the population, is the leading genetic risk factor of late‐onset Alzheimer's disease (AD), with *APOE4* homozygote (*APOE* 44) having more than 90% chance undergoing AD biology at 65 years old (Forta et al., Nature Medicine, 2024). Despite this near full penetrance, major gaps remain in our understanding of APOE4‐mediated neuropathology.

**Method:**

We compared human iPSC‐derived mixed cortical culture (hiMCC, mix of neurons and astrocytes) from *APOE* 44 and AD neutral *APOE3* allele carriers using proteomics and deeply sequenced transcriptomics data. We further validated our findings using *APOE* genotype CRISPR/Cas9‐edited isogenics hiMCC and postmortem brain multi‐omics data from large and highly phenotyped brain bank cohorts.

**Result:**

While transcriptomics and proteomics integration confirm impacts of APOE4 in extracellular matrix and lipid transport pathways, we found that *APOE4* induces in addition a robust reduction of the mRNA spliceosome machinery at protein level. In line with this, we further observed that *APOE4* induces important mRNA splicing defects, more specifically intron retention in genes regulating neuronal projection and are associated with concordant protein reduction. By analyzing scRNA‐seq data from isogenics hiMCC, we found that *APOE*4 induces this splicing defect specifically in a subpopulation of excitatory neurons with intense mRNA splicing activity for actin based projection. Finally, we confirmed these splicing defect with impact on neuronal projection genes observable also in APOE4 carrier postmortem brains even at early stage of disease progression, and found strong association with amyloid plaque and neurofibrillary tangles burden.

**Conclusion:**

Disruption of the excitatory neurons mRNA splicing activity for effective cell projection is one key element of APOE4 mediated neuropathology and appear early in the disease progression.

